# On the quest for selective constraints shaping the expressivity of the genes casting retropseudogenes in human

**DOI:** 10.1186/1471-2164-12-401

**Published:** 2011-08-08

**Authors:** Kamalika Sen, Soumita Podder, Tapash C Ghosh

**Affiliations:** 1 Bioinformatics Centre, Bose Institute, P 1/12, C.I.T. Scheme VII M, Kolkata- 700 054, India

**Keywords:** Expressivity, Protein intrinsic disorder, Connectivity, Alternative splicing, Protein stability, mRNA decay rate, Evolutionary rate

## Abstract

**Background:**

Pseudogenes, the nonfunctional homologues of functional genes are now coming to light as important resources regarding the study of human protein evolution. Processed pseudogenes arising by reverse transcription and reinsertion can provide molecular record on the dynamics and evolution of genomes. Researches on the progenitors of human processed pseudogenes delved out their highly expressed and evolutionarily conserved characters. They are reported to be short and GC-poor indicating their high efficiency for retrotransposition. In this article we focused on their high expressivity and explored the factors contributing for that and their relevance in the milieu of protein sequence evolution.

**Results:**

We here, analyzed the high expressivity of these genes configuring processed or retropseudogenes by their immense connectivity in protein-protein interaction network, an inclination towards alternative splicing mechanism, a lower rate of mRNA disintegration and a slower evolutionary rate. While the unusual trend of the upraised disorder in contrast with the high expressivity of the proteins encoded by processed pseudogene ancestors is accredited by a predominance of hub-protein encoding genes, a high propensity of repeat sequence containing genes, elevated protein stability and the functional constraint to perform the transcription regulatory jobs. Linear regression analysis demonstrates mRNA decay rate and protein intrinsic disorder as the influential factors controlling the expressivity of these retropseudogene ancestors while the latter one is found to have the most significant regulatory power.

**Conclusions:**

Our findings imply that, the affluence of disordered regions elevating the network attachment to be involved in important cellular assignments and the stability in transcriptional level are acting as the prevailing forces behind the high expressivity of the human genes configuring processed pseudogenes.

## Background

Pseudogenes often exemplified as 'genetic fossils' provide snapshots of evolutionary history of human genome [1]. Understanding the structural and functional attributes of the genes configuring pseudogenes by duplication and reverse transcription is now enlightening the research on these naturally occurring mutant genes in the frame of evolutionary studies supporting neutral mutation hypotheses [2]. The occurrence of these faulty replicates of normal genes in a genome is still a confounding matter. The processed or retropseudogenes [3] speculated as fossilized footprints of their parental gene expression [4] has become of increasing interest in the field of pseudogene evolution and comparative genomics since a burst of processed pseudogene genesis was observed early in primate evolution [5]. This kind of pseudogenes resembles the mature mRNA transcript of their functional counterpart. The processed transcript of a functional gene is reverse transcribed and integrated into a staggered chromosome break, followed by DNA synthesis and repair [6]. The process of reverse transcription and insertion is guided by the enzymatic machinery of LINE1 non-LTR retrotransposons [7]. Being derived from a mature mRNA product they lack the upstream promoters and are often entitled as "dead on arrival" [8] because of their acquired nonfunctionality [9] immediately upon the reinsertion process. Their structural feature shows a complete lack of introns, small flanking direct repeats and polyadenilation at the 3'-end [10].

During the last several years processed pseudogenes are being catalogued and characterized in many completely sequenced genomes including human. But there are very few reports on the structural and functional characterization of the human genes configuring this kind of pseudogenes. The pioneering work of Goncalves et al [11] focused on 181 human functional genes giving rise to 249 retropseudogenes. Their analysis revealed out some important features of the genes generating retropseudogenes regarding their evolutionary impact and structural attributes. They reported them (genes with retropseudogenes) to execute a significantly higher value of tissue distribution breadth than the genes lacking retropseudogenes. The preponderance of processed pseudogenes in housekeeping genes is also relevant to their higher expression level [12] than the genes without pseudogenes. Again, expression of a gene has been considered as a crucial marker of the evolutionary perseverance of the same till date [13]. So in the context of human processed pseudogene ancestor evolution gene expression and its different facets surely craves an intensive attention and a comprehensive discussion.

In this communication we probed the different aspects of gene expression of the human processed pseudogene ancestors. We characterized the high expressivity of the progenitor genes by their involvement in protein-protein interaction network, affluence of intrinsically unstructured protein regions, selection for alternative splicing technique, transcript stability and evolutionary conservation.

## Results

### Expression profile of the progenitor of processed pseudogenes and their involvement in protein-protein interaction network

Earlier it was reported that retropseudogene ancestors are predominantly housekeeping in nature showing a wide tissue distribution breadth [11]. The ubiquitously expressed housekeeping genes are also seen to execute high gene expression level [14]. We thus verified the high expressivity of the progenitors of processed pseudogenes (GFPψ genes) which are known to belong to housekeeping gene class. In our search the processed pseudogene ancestors exhibited significantly higher signal intensity (P = 1.61 × 10^-86 ^in Mann-Whitney test (M-W test), average expression level 2157.7995 [GFPψ genes], 961.2597 [GLψ genes]) as well as a higher EST count (P = 3.26 × 10^-136 ^in M-W test, average values 40.996 [GFPψ genes], 18.202 [GLψ genes]) expressing the mRNA abundance than the genes lacking pseudogenes. Now connectivity in interaction network and the gene expression level have been reported to be elevated for the genes belonging to housekeeping class [15]. Comparing the network attachment of the gene groups we observed a significantly higher value of interacting partners (P = 5.0 × 10^-3 ^in M-W test, average connectivity 8.3638 [GFPψ genes], 7.6434 [GLψ genes]) for the genes giving rise to processed pseudogenes than the genes with no pseudogenes. A significant positive correlation linking connectivity (between interacting partners) and signal intensity as well as connectivity and mRNA abundance was also obtained in our study (Table-1). Previous studies revealed that genes encoding hub proteins tend to be expressed with higher intensity [16]. Owing to this fact we also checked out the propensity of hub-protein encoding genes in our dataset and there we observed a significant predominance (Z score = 3.842, confidence level = 99%) of hub-protein encoding genes in the GFPψ genes (41.70%) than that of the GLψ genes (35.58%).

**Table 1:** Spearman's Rho and P values of the statistical correlations between the parameters analyzed in GFPψ genes and GLψ genes

Parameters	Gene expression (using Microarray)		Gene expression (using EST)	
	**ρ**	**P**	**ρ**	**P**

Interacting partners	0.145	1.0 × 10^-6^	0.121	1.0 × 10^-6^

Protein intrinsic disorder	0.058	1.84 × 10^-6^	0.039	2.94 × 10^-4^

mRNA decay rate	-0.118	1.0 × 10^-6^	-0.119	1.0 × 10^-6^

Evolutionary rate	-0.133	1.0 × 10^-6^	-0.137	1.0 × 10^-6^

### Predominance of disordered residues in the protein sequences of the genes configuring retropseudogenes

Proteins expressed at higher levels are stated to contain less disordered regions [17]. Hence, the ancestor genes of the processed pseudogenes which are seen to be highly expressed in our study were expected to show a low content of disordered residues. But surprisingly, in our analysis the progenitors of processed pseudogenes displayed a significantly higher amount of disordered residues (P = 6.56 × 10^-26 ^in M-W test, average disordered residue 41.924% [GFPψ genes], 32.991% [GLψ genes]) than the genes without pseudogenes and the percentage of disordered residues executed a significant positive association with the expression level of the two gene groups concerned (Table-1). To resolve this contradiction, we concentrated on the arguments which claimed that, the highly expressed proteins engaged in binding functions employ disordered regions to prevent aggregation [17] and the presence of unstructured regions is a constitutive feature of the hub-proteins since the disorder can act as a determinant of protein interactivity [18]. So, the GFPψ genes with higher expression level exhibited an elevated disorder due to their intense connectivity and higher propensity of hub-protein forming genes. To endorse our observations regarding the protein disorder we looked into other genomic and functional features of the retropseudoegene ancestors dealing with protein unfolded ness.

Earlier studies revealed an enrichment of disorder producing residues in highly stable proteins [19,20] due to the possibility that in vivo the disordered regions are no longer "unstructured" and are protected by binding to their biological targets. In our search the progenitors of the processed pseudogenes exhibit a significantly higher value of protein stability index (P = 2.40 × 10^-8 ^in M-W test, average value 3.658 [GFPψ genes], 3.390 [GLψ genes]) over the genes without pseudogenes.

Along with the protein stability and network involvement, we also probed into the genomic and functional features of the GFPψ genes to demonstrate their high disorder. Tandem repeat regions are seen to be prevalent in intrinsically unstructured regions [21]. We thus analyzed the presence of tandem repeat sequences in the human genes giving rise to processed pseudogenes which are affluent with disordered regions. Analyses revealed a significantly higher propensity (Z score = 2.558, confidence level = 95%) of genes having tandem repeat sequences in the aforementioned gene pool compared to that of the gene group lacking pseudogenes.

Researchers also provided evidence for the fact that proteins which are entirely disordered (80% to 100% disordered residue) with high level of expression can bypass the route of rapid degradation to successfully carry out their functions [19]. This kind of genes typically works as parts of large ribosomal subunits involved in transcription machinery [19]. In our experimentation, the genes with 80%-100% disordered residues and representing large ribosomal subunits engaged in transcription are significantly predominant (Z score = 9.819, confidence level = 99%, 11.28% [GFPψ genes], 4.16% [GLψ genes] and Z score = 13.05, confidence level 99%, 9.48% [GFPψ genes], 3.91% [GLψ genes] respectively) in the gene pool of the processed pseudogene ancestors than the pseudogene lacking gene set. Thus the processed pseudogene ancestors simultaneously exhibiting high expressivity and high disorder ness belong to a typical class of genes having the functional constraint to perform transcription associated works.

### The progenitors of processed pseudogenes produce a large number of spliced isoforms and execute a high level of mRNA stability

**Figure 1:**
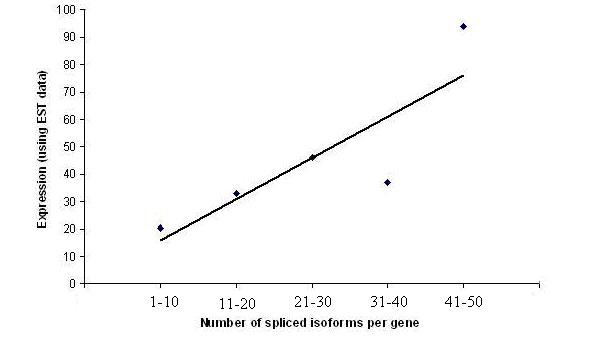
**Scatter plot showing correlation between number of spliced isoforms per gene and expression (using EST data)**.

Studies on genes with disordered residues revealed that alternative splicing sites are prevalent within the regions which are intrinsically unstructured [22]. Again it was established that alternative splicing can modulate gene expression by controlling transcript stability and translational efficacy [23]. In our search the genes with natively unfolded regions, giving rise to retropseudogenes are found to produce a number of spliced isoforms. We observed the number of those spliced isoforms to be significantly greater (P = 3.38 × 10^-4 ^in M-W test, average spliced isoform number 5.96 [GFPψ genes], 5.75 [GLψ genes] and respectively) in the aforementioned gene group than that of the genes lacking pseudogenes. There is also a significant positive correlation (Spearman's ρ = 0.357, P = 1.0 × 10^-6^) with the number of splicing isoforms and the mRNA abundance in the GFPψ gene group (Figure 1).

**Figure 2:**
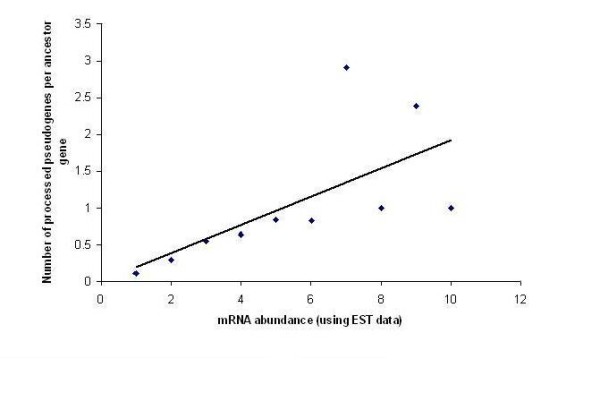
**Scatter plot showing correlation between mRNA abundance and number of processed pseudogenes per ancestor gene**. The EST data is clustered into 10 bins having a regular increase of mRNA abundance number 25.

Moreover, it was argued that, abundant mRNAs are likely to be considered as substrate of reverse-transcriptase enzyme [11]. As a consequence of the increased probability of reverse transcription process the chance for retro-pseudogenization event may also get raised which is reflected in our result obtained for GFPψ genes where we got a significantly positive correlation (Spearman's ρ = 0.231, P = 1 × 10^-6^) between mRNA abundance and the number of processed pseudogenes per ancestor gene (Figure 2).

While treating gene expression level, we again looked into the issue where it was stated that a higher rate of mRNA decay can be considered as an indicator of the lower gene expressivity [19]. In addition, the translational robustness of the genes configuring retroposed human mRNAs is also explained by their resistance to nonsense-mediated RNA decay [24]. Here, in our analysis, the genes promoting processed pseudogenes exhibit a coherence of higher expressivity and lower mRNA decay rate. We observed a significantly higher mRNA decay rate (P = 1.21 × 10^-6 ^in M-W test, 1.479 × 10^-1 ^[GLψ genes], 1.094 × 10^-1^[GFPψ genes]) of the genes without pseudogenes than the genes casting processed pseudogenes along with a significant negative correlation associating the mRNA decay rate and gene expression level (Table-1). Thus the mRNA stability of the GFPψ genes also account for their higher expressivity.

### Amino-acid sequence conservation of the ancestors of processed pseudogenes

Evolutionary studies on pseudogenes revealed that they congregate mutations at an extremely higher rate uniformly over their entirety when compared with their functional counterpart [25]. In our previous report on the duplicated pseudogenes we observed their ancestor genes to evolve at a higher rate than the genes configuring functional genes [26]. On the other hand, it has been reported that, genes with retropseudogenes encounter a stronger selective pressure for amino acid sequence conservation than the genes without retropseudogenes [11]. Besides, the rate of evolution is observed to be modulated by the intensity of gene expression of vertebrate genome [27]. Even in yeast the highly expressed genes are seen to evolve slowly [28]. These observations induced us to have a comparative look into the pattern of evolutionary rate of the GFPψ genes and the GLψ genes and to justify the relation with gene expression level. Our results showing a significantly lower rate of evolution (P = 8.1 × 10^-11 ^in M-W test, 1.378 × 10^-1 ^[GLψ genes], 1.261 × 10^-1 ^[GFPψ genes]) for the progenitors of processed pseudogenes than the genes without pseudogenes along with a significant negative correlation between gene expression and rate of evolution (Table-1) confirm the above mentioned facts.

## Discussion

Previous reports unveiling the housekeeping character and evolutionary persistence of the progenitors of processed pseudogenes demand further delving on their pattern of gene expression. Our investigations on gene expression level revealed a high expressivity of the human genes casting processed pseudogenes over the genes lacking pseudogenes. We thereafter tried to scoop out the genomic and functional traits of the genes processing retropseudogenes to elucidate their high expressivity.

The intensity of protein expression and physical interactions are seen to be integrated in humans [29]. Reports also claimed a high expressivity for the genes well connected (hub-protein encoding genes) in protein-protein interaction network [30]. In our study the high expressivity and wide tissue distribution breadth shaping the interaction pattern of the parent genes of processed pseudogenes in protein-protein interaction network reflect the same issue since they are found to execute high interactivity along with a predominance of hub-protein encoding genes. The disparity appeared in the results when we observed the retropseudogene ancestors with high gene expression displayed an affluence of disordered regions. We thence, demonstrated the disorderness as a prerequisite of their intense network involvement. The unstructured regions in their translated forms facilitate the network connectivity since previous reports demonstrated structural disorderness as a common characteristic of hub proteins [18]. The GFPψ genes harboring disordered regions and displaying a high expressivity and network connectivity are also observed to retain their translated form in a stable configuration. This may be due to the fact that, the regions with structural disorder keep up the stability of proteins in vivo through the attachment with their corresponding target molecules [20]. While evaluating the existence of disordered regions in the proteins encoded by the GFPψ genes we put forward more evidences providing their genomic and functional features. In doing so we delved into the fact that, hub-proteins are enriched with disordered residues and sequence repeats to enlarge available surface area predisposing them for functioning via protein-protein interactome [31]. In our research on the ancestors of retropseudogenes, the abundance of genes encoding hub-proteins and repeat sequences also affirms the occurrence for protein disordered regions. In addition, a positive correlation (Spearman's ρ = 0.071, P = 1.0 × 10^-6^) between the mRNA abundance and the propensity of repeat sequence containing gene supports the fact that, tandem repeats in human genes can positively regulate the level of transcription [32]. Moreover, proteins encoded by the GFPψ genes are observed to configure large ribosomal subunits engaged in transcription associated jobs. Our result thus provides support for the idea that, proteins carrying disordered regions are able to perform some essential functions directly linked to their structural disorder [33]. Thus, even the lack of well-defined 3-D structure of the proteins encrypted by the GFPψ genes also represents their significance and functional importance in human protein interactome. A positive correlation (Spearman's ρ = 0.09, P = 1.0 × 10^-6^) observed between protein stability index and mRNA abundance further confirms the fact that, the highly disordered proteins can bypass rapid degradation pathway to allow them to perform their important cellular functions [19]. In addition, the analysis of the mRNA decay rates of the two gene group yields slower mRNA decay for the GFPψ genes supporting earlier reports which displayed mRNA turnover as a factor to coordinate gene expression level via transcriptional and translational regulation [19,34]. Again, it was argued that intrinsically unstructured regions of a polypeptide segment offer sites for alternative splicing as the disordered regions can tolerate functional or regulatory diversity without any disturbance in protein sequence [22]. Furthermore, as the alternative splicing event accounts for the quantitative and qualitative regulation of gene expression [24], we tried to explain the elevation in mRNA abundance (representing expression level of the gene groups) observed in the GFPψ genes in terms of their inclination towards alternative splicing mechanism. Hence, we here hypothesize that, the GFPψ genes go through an extensive alternative splicing event to form a number of spliced isoforms elevating the mRNA abundance level. The degree of mRNA abundance and their endurance together may contribute to enhance the level of gene expression of the GFPψ genes. We here, also speculate that the higher mRNA abundance contributes for an elevated reverse-transcription process which in turn increases the chance for retro-pseudogenization. Moreover, as gene expression level is known to constrain sequence evolution [35] in yeast, we here, analyzing human genes, also examined rate of protein evolution and observed amino acid sequence conservation for the genes promoting processed pseudogenes in human which again affirms their functional significance. Finally, from the linear regression analysis we can confer that, though all the factors we analyzed here control the expressivity of the GFPψ genes but all of them can not act as independent regulator of the same. It is evident from the analyses that, only the percentage of disordered residues (β = 0.194) and the mRNA decay rate (β = -0.079) can independently control the expressivity where the disorder influences the gene expression most significantly. We also performed our analysis excluding the ribosomal proteins and got the same trend in our results and confirmed that their (ribosomal proteins) evolutionary persistence, higher expression and higher disorder did not predispose the results (Additional file 1).

## Conclusions

Taken together, we summarize that, the regulatory factors constraining the expressivity of the human genes configuring processed pseudogenes are their connectivity in protein-protein interaction network, alternative splicing mechanism, slower rate of mRNA decay and evolutionarily conserved nature. While, the presence of intrinsically unstructured regions, which is attributed by the occurrence of genes containing repeat sequences, increased protein stability and the functional constraint to perform the transcription regulatory jobs, enriches the network connectivity to be involved in important cellular assignments and increase the stability in transcriptional level. Thus, our study on the human processed pseudogene ancestors, exposing the genomic imperatives constraining their expressivity as well as a new facet of their physical and functional attributes will expand the future studies on pseudogene forming genes on the scaffold of human genome evolution.

## Methods

### Human genes forming processed pseudogenes

Human processed pseudogene annotations were achieved from pseudogene database (Human pseudogenes, Build 57) (http://www.pseudogene.org/) [36]. In our analysis the human genes forming processed pseudogenes are allocated as GFPψ genes and the genes without pseudogenes are assigned as GLψ genes. The number of genes retrieved as GFPψ genes and GLψ genes are 2362 and 38,862 respectively. The corresponding gene sequences were obtained from ftp://ftp.ncbi.nih.gov/genomes/H_sapiens/.

### Gene microarray expression data

The gene expression profile data were extracted from Human Gene Atlas GNF1H, MAS5 dataset (http://biogps.org/) [14]. The signal intensities across 79 tissues were averaged and are considered as expression level for each gene represented by their corresponding probe id. The number of genes retrieved as GFPψ genes and GLψ genes were 2362 and 38,862 respectively. However, we performed our other analyses with the genes providing Microarray expression intensity and the number of genes (in Microarray expression dataset) for GFPψ genes and GLψ genes are 1443 and 10192 respectively (Additional file 2).

### Protein-protein interaction data

The number of interacting partners of the genes in our dataset was obtained from HPRD (Human Protein Reference Database), version 7, (http://www.hprd.org) [37] and the genes maintaining more than 5 interacting partners were assigned as hub proteins.

### Disorder prediction in human proteome

Disorder predictions were carried out using the program FoldIndex [38] implementing the prediction method of Uversky et al [39]. To reduce the rate of false positives, disordered regions containing at least 30 contiguous disordered residues were considered [40]. The fraction of disordered residues was calculated by taking the ratio of the number of disordered residues to the total number of residues in the protein.

### Measurement of protein stability indices

Data for protein stability indices were obtained from the stability measures done by Yen et al [20] in their global protein stability assay of more than 8,000 human proteins. We mapped the stability measures to our gene dataset.

### Retrieval of repeat sequence containing genes

Repeat regions of genes were found out using the program Tandem repeats finder [41]. The propensity of repeat region containing genes was calculated by considering the ratio of number of genes having repeat regions to the total number of genes in each dataset.

### Functional annotations of our gene sets

The functional information assigned by the GO annotations of the highly disordered proteins was obtained from the Go term data of Edwards et al [19]. We mapped the given Go annotations on our dataset.

### Retrieval of alternatively spliced isoforms

Data for alternatively spliced isoforms [42] for the genes in dataset was downloaded from the ASD database (http://www.ebi.ac.uk/asd) containing splice patterns of human alternatively spliced genes.

### Measurement of mRNA abundance

mRNA abundance of the genes in our dataset was calculated using EST data attained from DFCI Gene Indices (http://compbio.dfci.harvard.edu/tgi/). Gene expression level was estimated calculating the number of occurrence of each gene among EST sequences from 179 cDNA libraries sampled with at least 10,000 ESTs [43,44]. Eliminating pathogenic and cancerous libraries 41 libraries were kept and alignments were made between the coding sequences of the gene groups with the EST dataset using BLASTN program with a sequence matching criterion of 60% identity and 80% overlaps. The overall EST count for each gene across 41 EST libraries represents their mRNA abundance.

### Evaluation of mRNA decay rates

mRNA decay rates of the genes in our dataset were retrieved from the report/analysis of Yang et al [45] where they measured the mRNA decay rates of 5,245 human transcript.

### Data for gene evolutionary rates

Evolutionary rates of the human genes (Homo sapiens (GRCh37)) in our dataset (genes with processed peudogenes and without pseudogenes) were achieved from Ensembl 58 (http://www.ensembl.org/biomart/martview) [46].

### Retrieval of genes encoding Ribosomal Proteins

Genes coding ribosomal proteins were obtained from Ribosomal Protein Gene Database (http://ribosome.miyazaki-med.ac.jp) [47].

## Abbreviations

**GFPψ genes: **Genes Forming Processed Pseudogenes; **GLψ genes: **Genes Lacking Pseudogenes.

## Authors' contributions

KS conceived of the study, collected the data, performed steps of bioinformatic analysis, statistical analysis and wrote the manuscript. SP contributed in the design of the study, collection of data and coordination. TCG supervised the study and helped to draft the manuscript. All authors read and approved the final manuscript.

## Supplementary Material

Additional file 1 **P values in Mann-Whitney tests for the comparative study performed between GFPψ genes and GLψ genes after removing all genes coding Ribosomal proteins from both the datasets**. Click here for file

Additional file 2 **Ensmble id of Genes Forming Processed Pseudogenes (GFPψ genes) and Genes Lacking any Pseudogenes (GLψ genes)**. Click here for file
