# Supplementary material for: On the quest for selective constraints shaping the expressivity of the genes casting retropseudogenes in human
Source: BMC Genomics. 2011 Aug 8;12:401. doi: 10.1186/1471-2164-12-401 (PMC3162935; doi:10.1186/1471-2164-12-401)
Supplement: Additional file 1 — P values in Mann-Whitney tests for the comparative study performed between GFPψ genes and GLψ genes after removing all genes coding Ribosomal proteins from both the datasets. [file 1471-2164-12-401-S1.DOC]

| Parameters | P value in M-W test |
| --- | --- |
| Microarray expression | 7.12 x 10^-68^ |
| EST count | 1.86 x 10^-107^ |
| Connectivity | 1 x 10^-3^ |
| % of disorder residues | 1.48 x 10^-21^ |
| Protein stability index | 2.18 x 10^-9^ |
| Alternatively spliced isoform Number | 2x 10^-3^ |
| mRNA decay | 1.06 x 10^-5^ |
| Evolutionary rate | 3.42 x 10^-7^ |
